# Gefitinib and Erlotinib Lead to Phosphorylation of Eukaryotic Initiation Factor 2 Alpha Independent of Epidermal Growth Factor Receptor in A549 Cells

**DOI:** 10.1371/journal.pone.0136176

**Published:** 2015-08-19

**Authors:** Satoshi Koyama, Tomohiro Omura, Atsushi Yonezawa, Satoshi Imai, Shunsaku Nakagawa, Takayuki Nakagawa, Ikuko Yano, Kazuo Matsubara

**Affiliations:** Department of Clinical Pharmacology and Therapeutics, Kyoto University Hospital, Kyoto, Japan; University of Parma, ITALY

## Abstract

Gefitinib and erlotinib are anticancer agents, which inhibit epidermal growth factor receptor (EGFR) tyrosine kinase. Interstitial lung disease (ILD) occurs in patients with non-small cell lung cancer receiving EGFR inhibitors. In the present study, we examined whether gefitinib- and erlotinib-induced lung injury related to ILD through endoplasmic reticulum (ER) stress, which is a causative intracellular mechanism in cytotoxicity caused by various chemicals in adenocarcinomic human alveolar basal epithelial cells. These two EGFR inhibitors increased Parkinson juvenile disease protein 2 and C/EBP homologous protein mRNA expressions, and activated the eukaryotic initiation factor (eIF) 2α/activating transcription factor 4 pathway without protein kinase R-like ER kinase activation in A549 cells. Gefitinib and erlotinib caused neither ER stress nor cell death; however, these agents inhibited cell growth via the reduction of cyclin-D1 expression. Tauroursodeoxycholic acid, which is known to suppress eIF2α phosphorylation, cancelled the effects of EGFR inhibitors on cyclin-D1 expression and cell proliferation in a concentration-dependent manner. The results of an EGFR-silencing study using siRNA showed that gefitinib and erlotinib affected eIF2α phosphorylation and cyclin-D1 expression independent of EGFR inhibition. Therefore, the inhibition of cell growth by these EGFR inhibitors might equate to impairment of the alveolar epithelial cell repair system via eIF2α phosphorylation and reduced cyclin-D1 expression.

## Introduction

Epidermal growth factor receptor (EGFR) tyrosine kinase inhibitors, such as gefitinib and erlotinib, are oral molecule-targeted drugs for non-small cell lung cancer. These drugs occasionally induce interstitial lung disease (ILD), especially interstitial pneumonia, as a critical adverse reaction [1, 2]. ILD patients with chest-imaging portraying ground-glass opacity and severe breathlessness have to discontinue therapies with these EGFR inhibitors. However, little is known about the pathogenesis, diagnosis, and treatment of such drug-induced ILD. The observed increase in ILD risk with gefitinib treatment has been shown to be higher in elderly smokers with preexisting ILD or poor performance status [3]. Alveolar type-II epithelial cells are believed to be progenitor cells in lung tissues. With injured alveoli, type-II cells proliferate and differentiate into type-I cells, leading to alveolus repair [4]. Thus, deterioration of this repair pathway in alveoli can be interpreted as a possible mechanism for the promotion of ILD in EGFR inhibitor therapy.

EGFR inhibitors reduce cyclin-D1 levels inducing cell cycle arrest in the G1 phase [5–7]. Phosphorylation of eukaryotic initiation factor (eIF) 2α reduces cyclin-D1 expression in mouse embryonic fibroblasts [8, 9], and is known to induce translational suppression [10, 11]. Namba *et al*. have reported that gefitinib may induce translational suppression of heat-shock protein 70 in adenocarcinomic human alveolar basal epithelial (A549) cells [12]. Phospho-eIF2α binds to and inhibits eIF2B, which converts eIF2-GDP into eIF2-GTP. This inhibition causes translational suppression as a result of the depletion of eIF2-GTP [10], which is one of the typical pathways under endoplasmic reticulum (ER) stress [11]. ER stress has been suggested to act as a causative factor in several lung injuries. Mutant surfactant protein C (L188Q), which is discovered in a kindred with familial interstitial pneumonia [13], incites ER stress in mice alveolar type-II epithelial cells [14]. ER stress mediates lung injury of some compounds, including cigarette smoke and herbicides (e.g. paraquat) [15, 16]. However, some of the molecule-targeted drugs, such as imatinib, sorafenib and dasatinib, have been reported to evoke ER stress [17–19]. Therefore, gefitinib and erlotinib may cause ER stress to induce lung injury related to ILD.

In the present study, we examined if pulmonary toxicity induced by EGFR inhibitors was associated with ER stress, using A549 cells as a model of human alveolar type II-like epithelial cells. We first investigated if alteration of signaling was associated with ER stress. We found that eIF2α was phosphorylated and cyclin-D1 was reduced in A549 cells treated with EGFR inhibitors. We further examined cytoprotective action of elF2α phosphorylation-suppressive tauroursodeoxycholic acid (TUDCA), and tested if this effect was EGFR-dependent.

## Materials and Methods

### Chemical Reagents

Gefitinib and erlotinib hydrochloride were obtained from LC Laboratories (Woburn, MA, USA) and Santa Cruz Biotechnology (Dallas, TX, USA), respectively. TUDCA and propidium iodide were commercially available from Sigma-Aldrich (St. Louis, MO, USA), while Hoechst 33342 and 3-(4,5-dimethyl-2-thiazolyl)-2,5-diphenyl-*2H*-tetrazolium bromide (MTT) were products of Dojindo Laboratories (Kumamoto, Japan). Tunicamycin and other chemical reagents were obtained from Wako Pure Chemicals (Osaka, Japan).

### Antibodies

Antibodies for C/EBP homologous protein (CHOP) (#2895), cleaved caspase-3 (#9661), cyclin-D1 (#2926), EGFR (#2232), eIF2α (#9722), and phospho-eIF2α (Ser51) (#9721), protein kinase R-like ER kinase (PERK) (#3192) were purchased from Cell Signaling Technology (Danvers, MA, USA), and that for KDEL sequence (ADI-SPA-827-F) was from Enzo Life Sciences (Loerrach, Germany). Anti-activating transcription factor 4 (ATF4) (sc-200) and β-actin (AC-15) antibodies were products of Santa Cruz Biotechnology (Dallas, TX, USA) and Sigma-Aldrich (St. Louis, MO, USA), respectively.

### Cell Culture and Drug Treatment

A549 cells (ECACC, 86012804) and PC-9 cells (kindly gifted by Dr. Menju, Department of Thoracic Surgery, Kyoto University) were respectively cultured in Dulbecco's modified eagle's medium (DMEM) and RPMI 1640 supplemented with 10% fetal bovine serum (Gibco, Carlsbad, CA, USA) in a humidified atmosphere with 5% CO_2_ at 37°C. Drugs in serum-free medium (final DMSO concentration: < 0.1%) were added to cells seeded on 24-well (for MTT assay) or 6-well plates (for real-time PCR, western blotting analysis or staining).

### MTT Assay

Cells treated with/without drugs were incubated with 0.5 mg/mL MTT and was assayed for 30 min at 37°C. After discarding the medium, stained cells were dissolved in 1 mL of DMSO before the optical density was measured at 560 nm (reference wavelength, 630 nm).

### RNA Isolation and Quantitative Real-Time PCR

Total RNA was isolated from A549 cells with an RNeasy Plus Mini Kit (Qiagen, Hilden, Germany) according to manufacturer’s instructions. Complementary DNA (cDNA) was generated from RNA (20 μg) using a High Capacity RNA-to-cDNA Kit (Applied Biosystems, Carlsbad, CA, USA). Quantitative real-time PCR was performed according to the StepOnePlus Real-Time PCR System (Applied Biosystems) and TaqMan Fast Advanced Master Mix (Applied Biosystems). The probe-primer solutions specific for the following genes were used (obtained from Applied Biosystems): Binding immunoglobulin protein (*BIP*) (Hs00607129_gH), *CHOP* (Hs00358796_g1), HMG-CoA reductase degradation 1 (*HRD1*) (Hs00381211_m1), Parkinson juvenile disease protein 2 (*PARK2*) (Hs01038325_m1), suppressor or enhancer of lin-12 1 (*SEL1*) (Hs01071406_m1) and *18S rRNA* (Hs99999901_s1). The agent *18S rRNA* was used an internal control to normalize mRNA expression levels.

### Western Blotting Analysis

Cell samples collected in lysis buffer (20 mM HEPES, 120 mM NaCl, 5 mM EDTA, 1% Triton X-100, 10% glycerol, 10 mM NaF, 2 mM Na_3_VO_4_) were placed in protease inhibitor cocktail (Nacalai Tesque, Kyoto, Japan), and protein concentrations were determined using the Bradford assay. Equal amounts of total protein were separated by SDS-PAGE and then transferred to PVDF membrane. Blocking was performed at room temperature for 1 h in TBS-T with 5% skim milk (BD Falcon, Franklin Lakes, NJ), followed by overnight incubation with different primary antibodies (described above) in TBS-T at 4°C. Appropriate secondary antibodies were then used, and proteins were visualized using chemiluminescence (Luminata Crescendo, Millipore, Billerica, MA, USA; ECL select, GE Healthcare, Little Chalfont, UK). The intensities of protein levels, which were analyzed using Image J software from NIH, were corrected with the respective β-actin levels.

### Silencing of EGFR Expression

Stealth RNAi siRNA to human *EGFR* (HSS103116) and non-targeting stealth RNAi siRNA (negative control low GC duplex) (Invitrogen, Carlsbad, CA, USA) were seeded in 6-well plates 1 day before transferring A549 cells for transfection with 50 pmol of siRNA, 5 μL of lipofectamine RNAiMAX (Invitrogen) and 500 μL of Opti-MEM (Invitrogen). The transfected A549 cells were then tested with the respective drugs 3 or 48 h after treatment.

### Evaluation of Cell Death

After drug treatment, trypsinized cells collected by centrifugation (190 g, 5 min, room temperature) were re-suspended in 1×PBS. Trypan blue staining and cell-counting were performed with Countess (Invitrogen, Carlsbad, CA, USA), according to the manufacturer’s instruction. For detection of apoptotic and necrotic cells, an aliquot of 50 μL of cells was suspended with 50 μL of 1×Binding Buffer containing Hoechst 33342, Ethidium Homodimer III (EthD-III) and FITC-Annexin V. After standing for 15 min at room temperature followed by washing with 1×Binding Buffer, according to the instruction manual of Apoptotic/Necrotic/Healthy Cells Detection Kit (PromoKine, Heidelberg, Germany). The fluorescent images were captured and examined with BZ-9000 (Keyence, Osaka, Japan). Three independent photographs were taken at 100-fold magnification, and the stained cells were counted. Apoptotic and necrotic cells were estimated by [FITC-Annexin V] / [Hoechst 33342] and [EthD-III] / [Hoechst 33342], respectively.

### Statistical Analysis

Quantitative data are represented as means ± S.E.M. Data were statistically analyzed using one-way analysis of variance, followed by the Dunnett’s or Tukey-Kramer’s two-tailed test to evaluate differences between more than three groups. Probability values of less than 0.05 were considered statistically significant. Statistical analysis was performed using GraphPad Prism 5 (GraphPad Software, La Jolla, CA, USA).

## Results

### Gefitinib and Erlotinib Suppressed Cell Growth of A549 Cells

**Fig 1:**
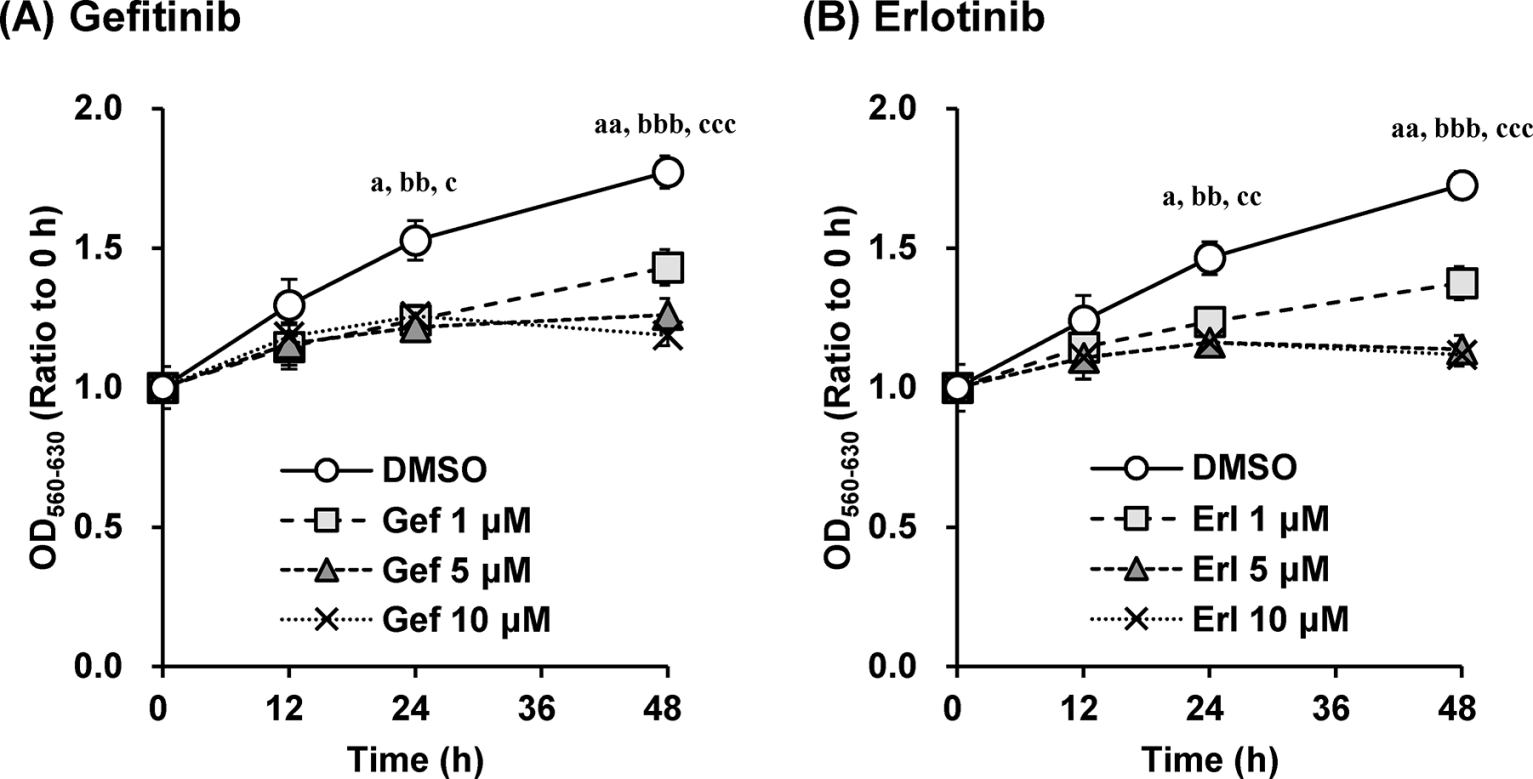
Gefitinib and erlotinib suppressed growth of A549 cells in a concentration-dependent manner. The effects of (A) gefitinib and (B) erlotinib (1–10 μM) on cell proliferation of A549 cells were investigated. Cell counts were estimated by the MTT assay. Data are expressed as means ± S.E.M. of three independent experiments. Each symbol indicates significant differences from DMSO group; a, *p* < 0.05; aa, *p* < 0.01 (DMSO vs. 1 μM), bb, *p* < 0.01; bbb, *p* < 0.001 (DMSO vs. 5 μM), c, *p* < 0.05; cc, *p* < 0.01; ccc, *p* < 0.001 (DMSO vs. 10 μM), one-way ANOVA with Dunnett’s post hoc tests.

Based on the effect of gefitinib and erlotinib on cell proliferation of A549 cells, the EGFR inhibitors suppressed proliferation of A549 cells in a concentration-dependent manner (Fig 1).

### Gefitinib and Erlotinib Induced the Up-Regulation of *PARK2* and *CHOP* mRNA in A549 Cells

**Fig 2:**
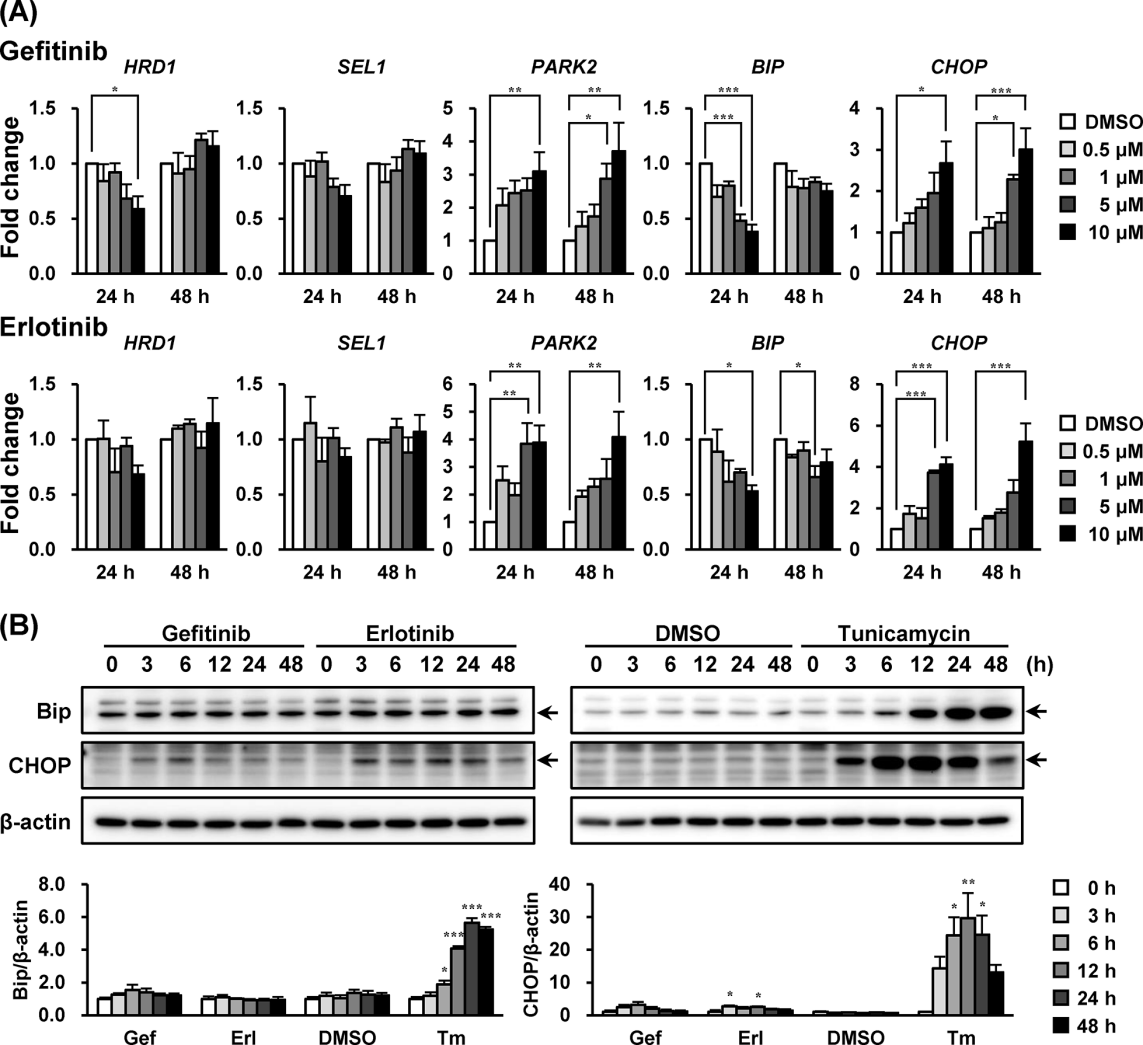
Gefitinib and erlotinib induced the up-regulation of *PARK2* and *CHOP* mRNA in A549 cells. The effects of gefitinib and erlotinib on (A) mRNA and (B) protein expression levels of unfolded protein response (UPR)-related genes in A549 cells were investigated. (A) A549 cells were treated with gefitinib or erlotinib (0.5–10 μM) for 24 and 48 h accordingly. Each mRNA expression level was normalized to *18S rRNA* level, and plotted relative to the control value (designated as 1.0). Data are expressed as means ± S.E.M. of at least three independent experiments. (B) A549 cells were treated with gefitinib (10 μM), erlotinib (10 μM) or tunicamycin (2.5 μg/mL) for indicated times. Representative images of three independent experiments are shown. Asterisks indicate significant differences from (A) DMSO or (B) 0 h group (*; *p* < 0.05, **; *p* < 0.01, ***; *p* < 0.001, one-way ANOVA with Dunnett’s post hoc tests).

Next, we investigated if unfolded protein response (UPR)-related molecules were influenced by EGFR inhibitors in A549 cells. After incubation with these drugs for 24 and 48 h in increasing concentrations, both gefitinib and erlotinib significantly increased the mRNA levels of *PARK2* (*P* < 0.01, 10 μM) and *CHOP* (*P* < 0.001, 10 μM) (Fig 2A), encoding as E3 ubiquitin ligase [20] and an apoptosis mediator [21, 22], respectively. The *BIP* mRNA level, where protein is the molecular chaperone induced by ER stress, was transiently decreased by exposure to gefitinib and erlotinib before being reversed to the baseline level without affecting mRNA expressions of *HRD1* (an E3 ubiquitin ligase) [23] and *SEL1* (an HRD1 stabilizer) [24].

At 10 μM, gefitinib and erlotinib transiently elicited a slight increase in CHOP levels without altering Bip levels during the assay (up to 48 h). Parkin protein encoded by *PARK2* gene was not detected (data not shown). Parkin in A549 cells could be under the detection limit because the expression might be low in these cells. We also confirmed that 2.5 μg/mL of tunicamycin (an ER stress inducer) substantially elevated CHOP and Bip protein levels in A549 cells (Fig 2B).

### Gefitinib and Erlotinib Decreased Cyclin-D1 without Activating Caspase-3

**Fig 3:**
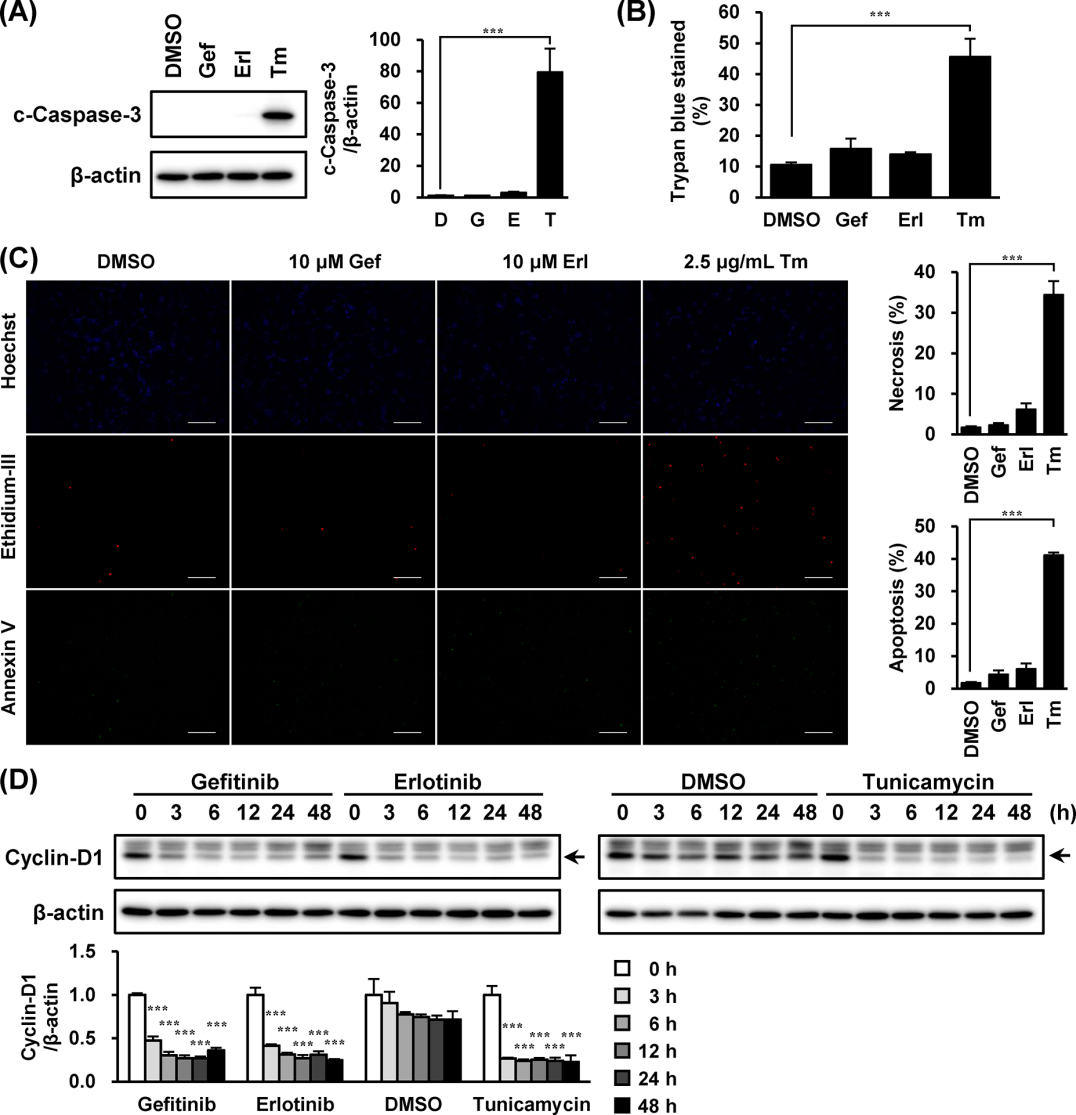
Gefitinib and erlotinib decreased cyclin-D1 without activation of caspase-3. A549 cells were treated with gefitinib (10 μM), erlotinib (10 μM) or tunicamycin (2.5 μg/mL) for 48 h or indicated times. (A) Whole-cell lysates were analyzed by immunoblotting using antibodies respectively specific against cleaved caspase-3 and β-actin. (B) Rates of trypan blue stained cells were estimated from three independent experiments and expressed as means ± S.E.M. (C) Cells were stained by Hoechst 33342, Ethidium-III and Annexin V (Magnification ×100, scale bar; 200 μm). (D) Whole-cell lysates were analyzed by immunoblotting using antibodies respectively specific against cyclin-D1 and β-actin. Representative images of three independent experiments are shown. Asterisks indicate significant differences from (A, B and C) DMSO or (D) 0 h group (***; *p* < 0.001, one-way ANOVA with Dunnett’s post hoc tests).

Because proapoptotic-signaling from CHOP leads to caspase-3 activation [25], we examined if EGFR inhibitors would activate caspase-3 in A549 cells. In accordance with CHOP alteration, cleaved caspase-3 was observed in cells treated with tunicamycin; however, such an event was not detected in cells treated with EGFR inhibitors (Fig 3A). Although tunicamycin evoked cell death in A549 cells, gefitinib and erlotinib did not affect the cells (Fig 3B and 3C). Meanwhile, treatment of these EGFR inhibitors reduced cyclin-D1 expression (Fig 3D).

### Gefitinib and Erlotinib Activated eIF2α/ATF4 Signaling

**Fig 4:**
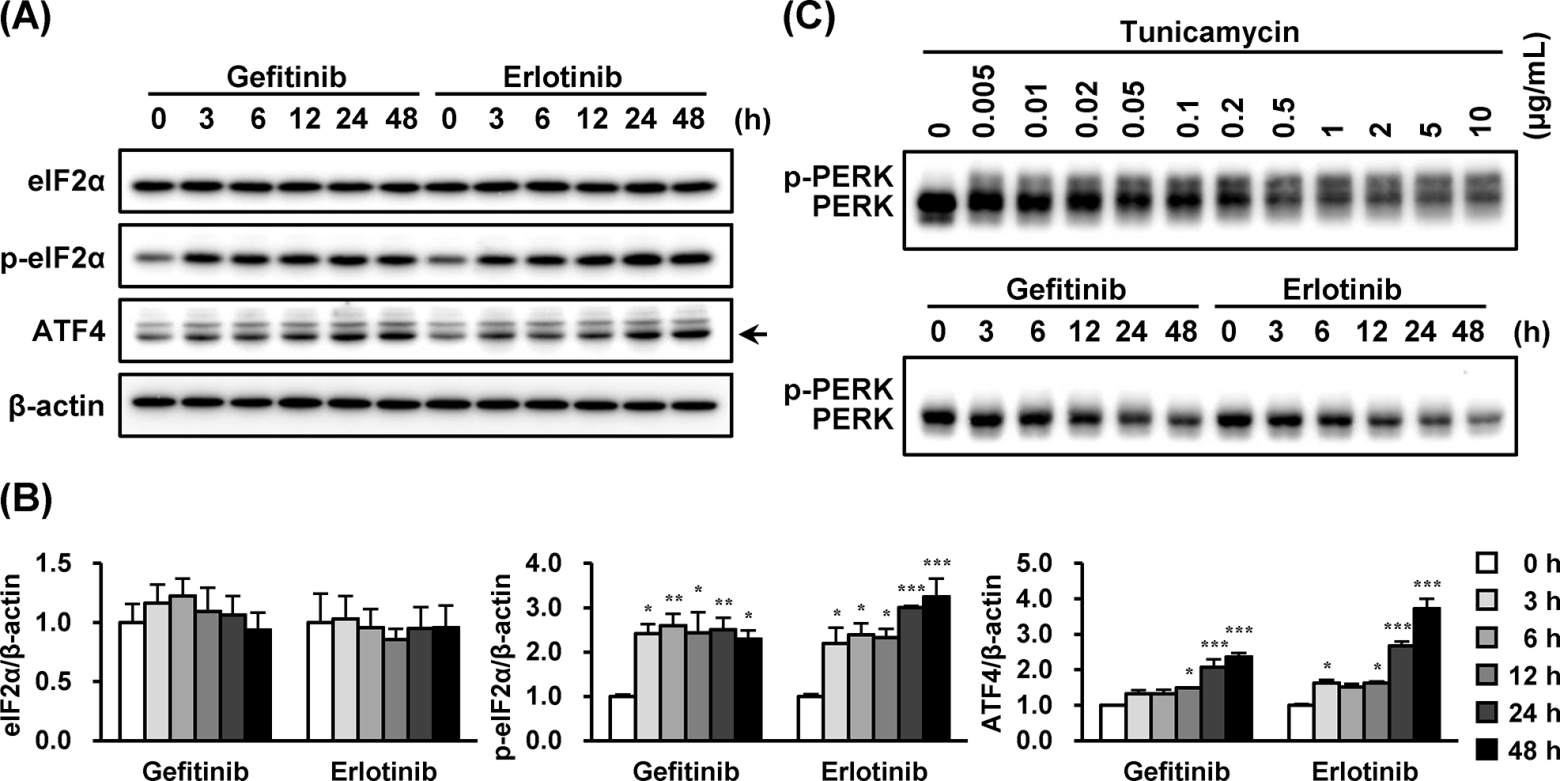
Gefitinib and erlotinib activated eIF2α/ATF4 signaling. The effects of gefitinib and erlotinib (10 μM) on (A) eIF2α/ATF4 activation and (C) PERK migration in A549 cells were investigated. A549 cells were treated with gefitinib (10 μM) or erlotinib (10 μM) for indicated times and tunicamycin (0.005–10 μg/mL) for 6 h. Whole-cell lysates were analyzed by immunoblotting using antibodies respectively specific against (A) eIF2α, phosphorylated eIF2α, ATF4, β-actin, and (C) PERK. Representative images of three independent experiments are shown. (B) Asterisks indicate significant differences from 0 h group (*; *p* < 0.05, **; *p* < 0.01, ***; *p* < 0.001, one-way ANOVA with Dunnett’s post hoc tests).

Because ATF4 analogously targets upstream of *CHOP* and *PARK2* genes [26, 27], we next investigated if EGFR inhibitors activated the PERK/eIF2α/ATF4 pathway. As shown in Fig 4A and 4B, ATF4 was induced and eIF2α was phosphorylated in A549 cells treated with EGFR inhibitors. In addition, ATF4 induction was observed to lag behind eIF2α phosphorylation. When PERK is phosphorylated in ER stress conditions, gel mobility of PERK shifts upward [28]. However, this phenomenon was not confirmed when A549 cells were exposed to EGFR inhibitors (Fig 4C).

### TUDCA Suppressions of eIF2α Phosphorylation and Cyclin-D1 Reduction by EGFR Inhibitors

**Fig 5:**
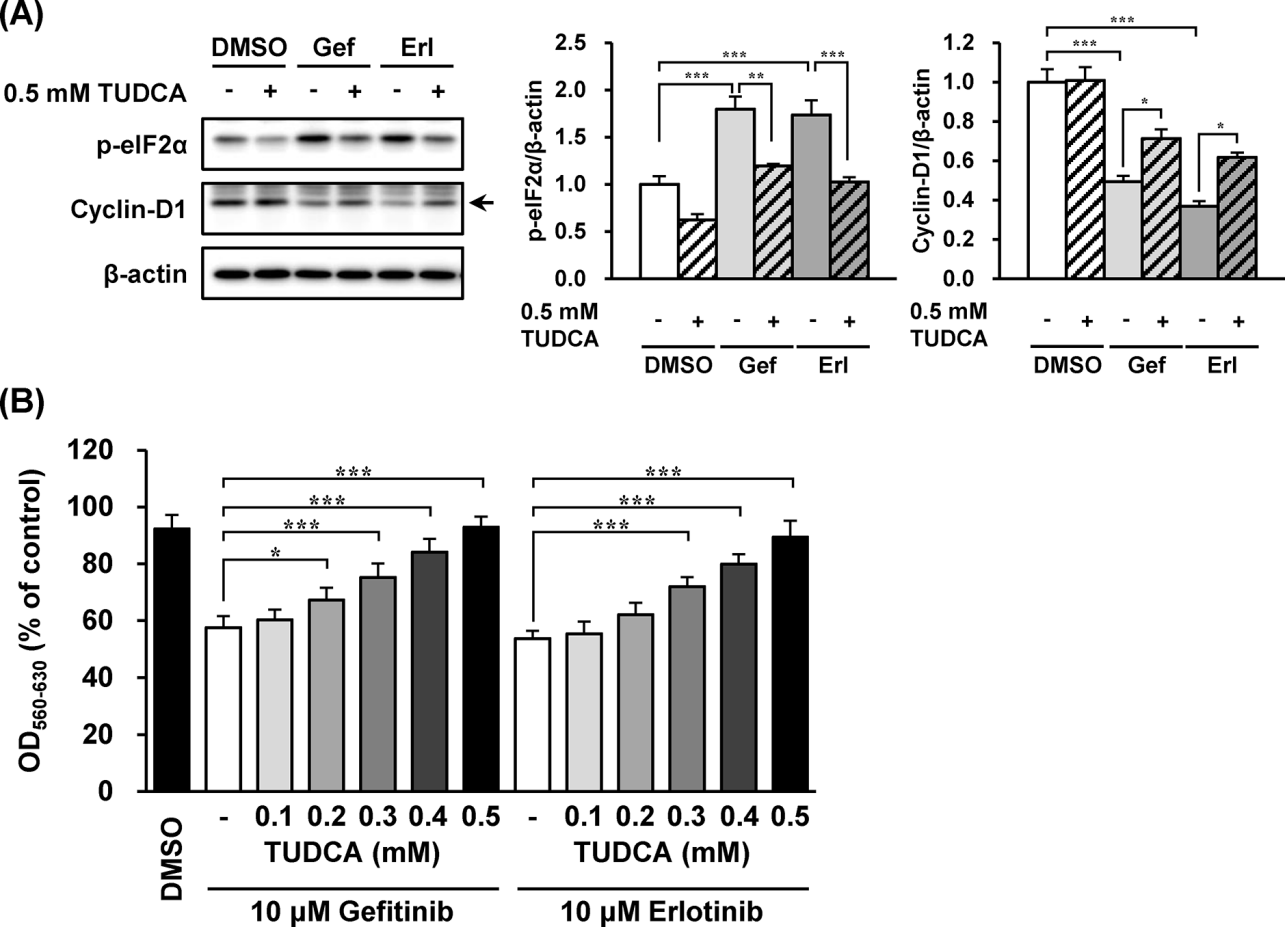
The eIF2α phosphorylation and cyclin-D1 reduction by EGFR inhibitors were suppressed by tauroursodeoxycholic acid (TUDCA). The effects of TUDCA on growth inhibition by EGFR inhibitors in A549 cells were investigated. (A) A549 cells were treated with gefitinib or erlotinib (10 μM) with/without TUDCA (0.5 mM) for 3 h (A) and 48 h (B). (A) Whole-cell lysates were analyzed by immunoblotting using antibodies respectively specific against phosphorylated eIF2α, cyclin-D1, and β-actin. Representative images of five independent experiments are shown. (B) Cell counts were estimated by the MTT assay. Data are expressed as means ± S.E.M. of three independent experiments. Asterisks indicate significant differences between two groups (*; *p* < 0.05, **; *p* < 0.01, ***; *p* < 0.001, one-way ANOVA with Tukey-Kramer’s tests).

As phosphorylation of eIF2α induces cyclin-D1 reduction [8, 9], the association of eIF2α phosphorylation with cyclin-D1 reduction caused by EGFR inhibitors was investigated. TUDCA, which is known to inhibit the phosphorylation of eIF2α [29, 30], suppressed eIF2α phosphorylation induced by gefitinib and erlotinib (Fig 5A). The reduction of cyclin-D1 expression by EGFR inhibitors was also alleviated by concomitant treatment with TUDCA (Fig 5A). In accordance with cyclin-D1 alteration by EGFR inhibitors, TUDCA suppressed the inhibition of cell growth induced by gefitinib and erlotinib (Fig 5B).

### Phosphorylation of eIF2α Induced by EGFR Inhibitors Was Independent of EGFR Inhibition

**Fig 6:**
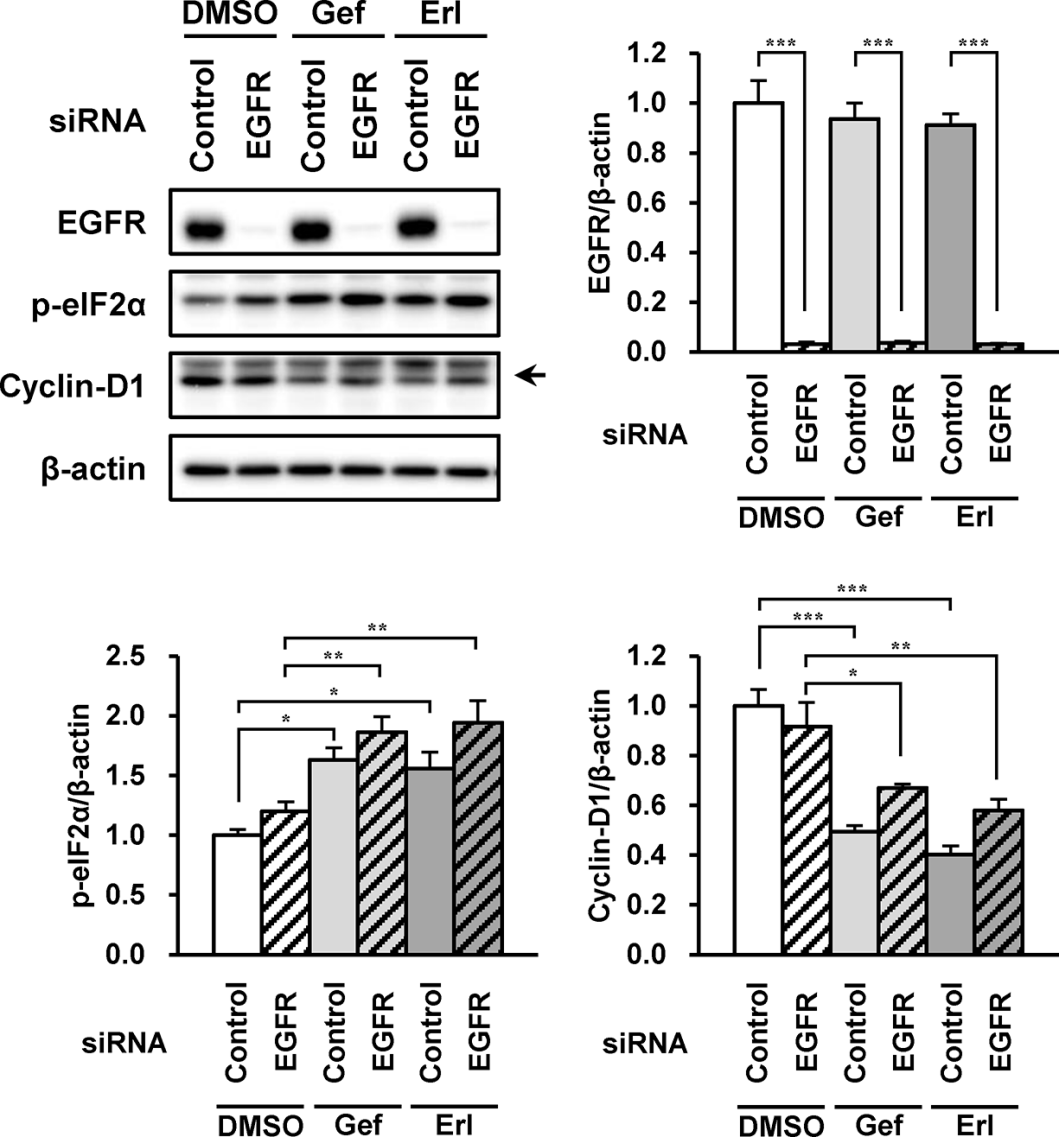
Phosphorylation of eIF2α induced by EGFR inhibitors was independent of EGFR inhibitory effect. Effects of EGFR knockdown on eIF2α phosphorylation and alteration of cyclin-D1 levels induced by gefitinib and erlotinib were investigated. A549 cells were transfected with 50 pmol of siRNA against *EGFR* or control siRNA for 48 h before treated with gefitinib or erlotinib (10 μM) for further 3 h. Whole-cell lysates were analyzed by immunoblotting using antibodies respectively specific against EGFR, phosphorylated eIF2α, cyclin-D1, and β-actin. Representative images of five independent experiments are shown. Asterisks indicate significant differences between two groups (*; *p* < 0.05, **; *p* < 0.01, ***; *p* < 0.001, one-way ANOVA with Tukey-Kramer’s tests).

Finally, we examined if eIF2α phosphorylation and cyclin-D1 reduction by EGFR inhibitors were due to their inhibitory effect against EGFR. *EGFR* siRNA strongly silenced EGFR expression without suppressing phosphorylation of eIF2α in A549 cells treated with the EGFR inhibitors (Fig 6). The reduction of cyclin-D1 induced by EGFR inhibitors did not fully recover (to baseline) by EGFR-silencing.

## Discussion

Since lung cancer patients often undergo various pulmonary toxic events, such as development of lung cancer and treatment with radiotherapy or chemotherapy, it is important for their epithelial cells to repair the alveoli in the lung [31]. As proliferation of human alveolar type II epithelial cells plays an important role in alveolar repair as progenitor cells [32], we examined if EGFR inhibitors (gefitinib and erlotinib used in this study) would affect the model human alveolar type II-like epithelial A549 cells in association with ER stress. Our findings revealed that both inhibitors suppressed proliferation of A549 cells in a concentration-dependent manner. Concurrent with growth inhibition, A549 cells exposed to these inhibitors increased mRNA expressions of *CHOP* and *PARK2* in this cell line. *CHOP* gene can be activated via the ER stress response element (ERSE) [33], unfolded protein response element (UPRE) [34], and amino acid response element (AARE). ATF4 targets at AARE [27], and transcriptionally regulates the *PARK2* gene [26]. We therefore investigated the effects of gefitinib and erlotinib on the PERK/eIF2α/ATF4 pathway by EGFR inhibitors in A549 cells. Our results demonstrated that these drugs induced phosphorylation of eIF2α and activation of ATF4, thus confirming that gefitinib and erlotinib (EGFR inhibitors) induced *CHOP* and *PARK2* mRNA expressions via activation of the eIF2α/ATF4 pathway.

Despite eliciting eIF2α phosphorylation, gefitinib and erlotinib did not activate PERK, which is one of the sensor molecules in ER stress [35, 36]. Additionally, these two drugs did not change the mRNA expressions of *HRD1* and *SEL1L*. Transcriptions of both *HRD1* and *SEL1* mRNA are regulated by UPRE and/or ERSE, which are the targets influencing the ATF6 and inositol requiring kinase 1α (IRE1α/X-box binding protein 1 pathway [37, 38]. From the speculative ‘calmness’ of these three sensors (PERK, IRE1α and ATF6) responsive to ER stress, our results indicate that gefitinib and erlotinib would not induce ER stress response in A549 cells. Since eIF2α has been reported to be phosphorylated not only by PERK, but also by general control non-derepressible-2 [39], RNA-activated protein kinase R [40], and heme-regulated inhibitor [41], examination of the effects of these EGFR inhibitors on the aforesaid molecules are warranted.

Although CHOP protein was transiently and slightly increased by the EGFR inhibitors tested here, the fact that CHOP induction precedes ATF4 induction suggests that this CHOP protein increase was probably not mediated by ATF4. Moreover, phosphorylation of eIF2α preferentially enhances *CHOP* mRNA translation mediated by an upstream open-reading frame through intracellular eIF2-GTP depletion [42]. As such, a mechanism involving eIF2-GTP depletion to enhance *CHOP* mRNA translation after eIF2α phosphorylation induced by these EGFR inhibitors may be plausible.

In this study, gefitinib and erlotinib did not induce caspase-3 activation when an increase in CHOP was observed. An adequate CHOP expression level can actually beget apoptosis in cells [43]. However, the induction of CHOP by gefitinib and erlotinib was much lower than that by tunicamycin (which induced significant cell death). Alternatively, persistent translational suppression by phospho-eIF2α might have prevented downstream mRNA of CHOP being translated into protein production. When exposed to concomitant treatment with TUDCA and EGFR inhibitors, the repression of phospho-eIF2α levels was observed coincidently with an alleviation of cyclin-D1 decrease, indicating that EGFR inhibitors reduced cyclin-D1 expression via eIF2α phosphorylation. Furthermore, when TUDCA prevented cyclin-D1 reduction induced by the EGFR inhibitors, growth inhibition was not detected. These results suggest that TUDCA might have promoted alveolar repair by preventing EGFR inhibitors from suppressing type-II cell growth. Although both drugs also phosphorylated eIF2α in PC-9 cells, which are human lung cancer cell line and are extremely sensitive to EGFR inhibitors, the protective effect of TUDCA was not found in this cell line (S1 and S2 Figs), suggesting that TUDCA treatment would not disturb the anti-cancer efficacy of EGFR inhibitors in non-small cell lung cancer patients. In this study, gefitinib and erlotinib did not induce apoptosis or necrosis in A549 cells. Since resistance of A549 cells to EGFR inhibitors is due to v-Ki-ras2 Kirsten rat sarcoma viral oncogene homolog (KRAS) mutation, the present data obtained from this cell line do not inform whether these drugs induce cell death in normal lung cells, which express normal KRAS. This is the limitation of our study to clarify the cellular mechanism of pulmonary toxicity related to ILD using A549 cells.

Finally, we demonstrated in the siRNA study that eIF2α phosphorylation induced by gefitinib and erlotinib was observed not to involve EGFR function. This result strongly supports the view that the eIF2α phosphorylation occurred independent of the EGFR inhibitory action. In contrast, EGFR-silencing partly ameliorated the cyclin-D1 reduction by EGFR inhibitors, implying that the event could occur in a manner both dependent and independent of EGFR, possibly via the eIF2α phosphorylation pathways. Cyclin-D1 repression induced by EGFR inhibitors has been believed to depend on the EGFR inhibitory effect, because EGF-signaling is associated with the activation of signal transducer and activator of transcription 3 and mitogen-activated protein kinase, as well as direct induction of cyclin-D1 transcription via EGFR binding to the cyclin-D1 promoter [44–46]. However, our results suggest that the cyclin-D1 reduction caused by EGFR inhibitors also occurred via the non-EGFR pathway.

In conclusion, the EGFR inhibitors used in our study, gefitinib and erlotinib, induced eIF2α phosphorylation independently via EGFR inhibition. Followed by both EGFR inhibition and eIF2α phosphorylation, cyclin-D1 reduction raised growth inhibition of A549 cells (the model of alveolar epithelial cells). Because growth inhibition could be responsible for lung injury [47, 48], phosphorylation of eIF2α induced by these EGFR inhibitors may potentially contribute to repair inhibition of alveoli. However, further studies to clarify the mechanism of targeting eIF2α phosphorylation by EGFR inhibitors are warranted. The present findings may serve as a potential approach to preventing incidences of ILD and pulmonary fibrosis induced by EGFR tyrosine kinase inhibitors, such as gefitinib and erlotinib.

## Supporting Information

S1 Fig Gefitinib and erlotinib arised cell death of PC-9 cells. PC-9 cells were treated with gefitinib or erlotinib (10 μM) for indicated times (A and B) and 24 h (C). Cell counts were estimated by the MTT assay. Data are expressed as means ± S.E.M. of three independent experiments. Each symbol indicates significant differences from DMSO group; a, *p* < 0.05; aa, *p* < 0.01; aaa, *p* < 0.001 (DMSO vs. 0.01 μM), bbb, *p* < 0.001 (DMSO vs. 0.1 μM), ccc, *p* < 0.001 (DMSO vs. 1 μM), ddd, *p* < 0.001 (DMSO vs. 10 μM), one-way ANOVA with Dunnett’s post hoc tests. (C) Cells were stained by Hoechst 33342, Ethidium-III and Annexin V (Magnification ×100, scale bar; 200 μm). Asterisks indicate significant differences from DMSO group. (***; *p* < 0.001, one-way ANOVA with Dunnett’s post hoc tests).
(TIFF)

S2 Fig Gefitinib and Erlotinib phosphorylated eIF2α in PC-9 cells. PC-9 cells were treated with gefitinib or erlotinib (10 μM) with/without TUDCA for indicated times (A), 3 h (B) and 48 h (C). Whole-cell lysates were analyzed by immunoblotting using antibodies respectively specific against phosphorylated eIF2α, cyclin-D1 and β-actin. Representative images of three independent experiments are shown. (C) Cell counts were estimated by the MTT assay. Data are expressed as means ± S.E.M. of three independent experiments. Asterisks indicate significant differences between two groups. (*; *p* < 0.05, **; *p* < 0.01, ***; *p* < 0.001, one-way ANOVA with Tukey-Kramer’s tests).
(TIFF)
