# Supplementary figures and images for: Gefitinib and Erlotinib Lead to Phosphorylation of Eukaryotic Initiation Factor 2 Alpha Independent of Epidermal Growth Factor Receptor in A549 Cells
Source: PLoS One. 2015 Aug 19;10(8):e0136176. doi: 10.1371/journal.pone.0136176 (PMC4545731; doi:10.1371/journal.pone.0136176)

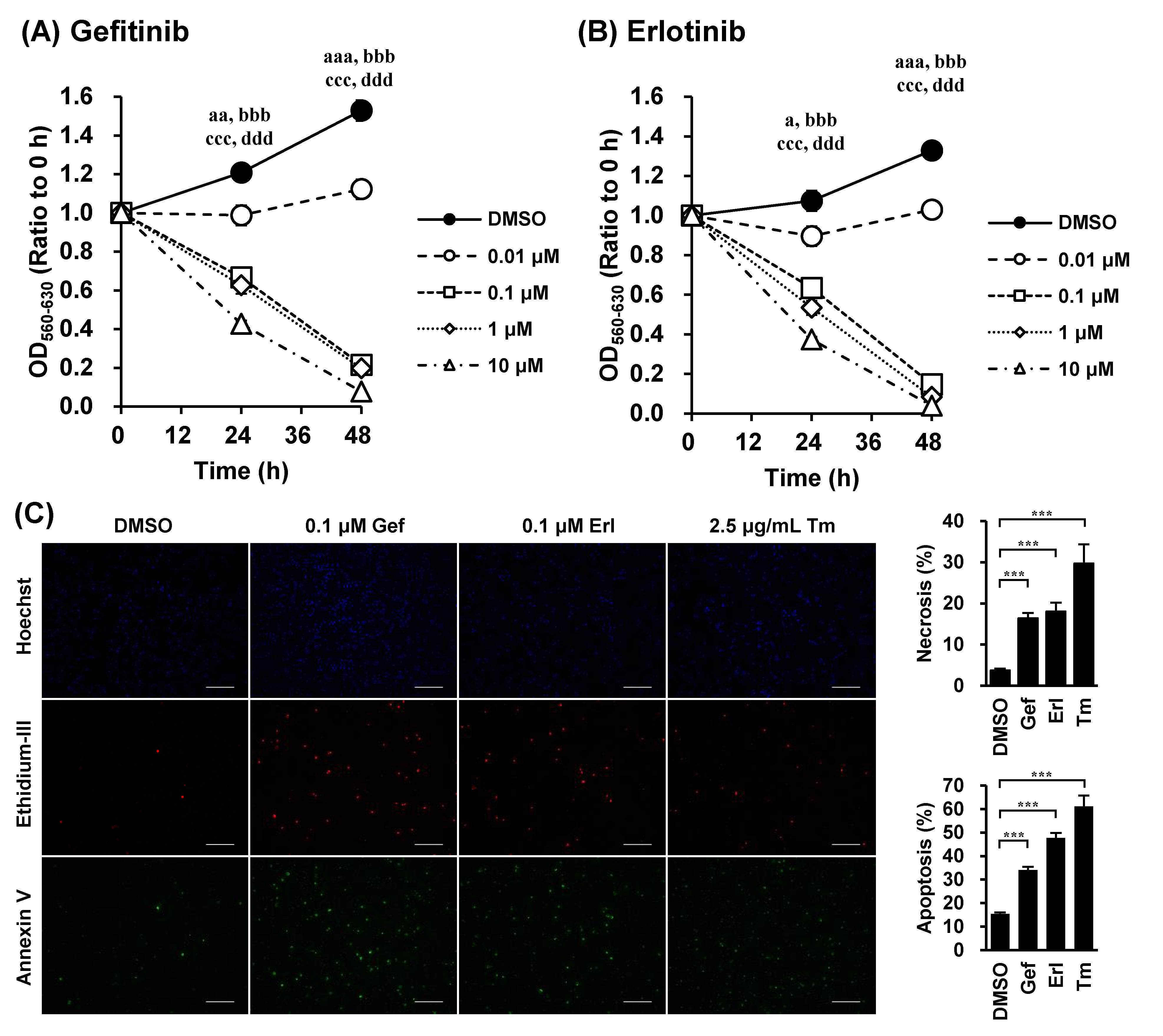

Supplement: S1 Fig — PC-9 cells were treated with gefitinib or erlotinib (10 μM) for indicated times (A and B) and 24 h (C). Cell counts were estimated by the MTT assay. Data are expressed as means ± S.E.M. of three independent experiments. Each symbol indicates significant differences from DMSO group; a, p < 0.05; aa, p < 0.01; aaa, p < 0.001 (DMSO vs. 0.01 μM), bbb, p < 0.001 (DMSO vs. 0.1 μM), ccc, p < 0.001 (DMSO vs. 1 μM), ddd, p < 0.001 (DMSO vs. 10 μM), one-way ANOVA with Dunnett’s post hoc tests. (C) Cells were stained by Hoechst 33342, Ethidium-III and Annexin V (Magnification ×100, scale bar; 200 μm). Asterisks indicate significant differences from DMSO group. (***; p < 0.001, one-way ANOVA with Dunnett’s post hoc tests). (TIFF) [file pone.0136176.s001.tiff]

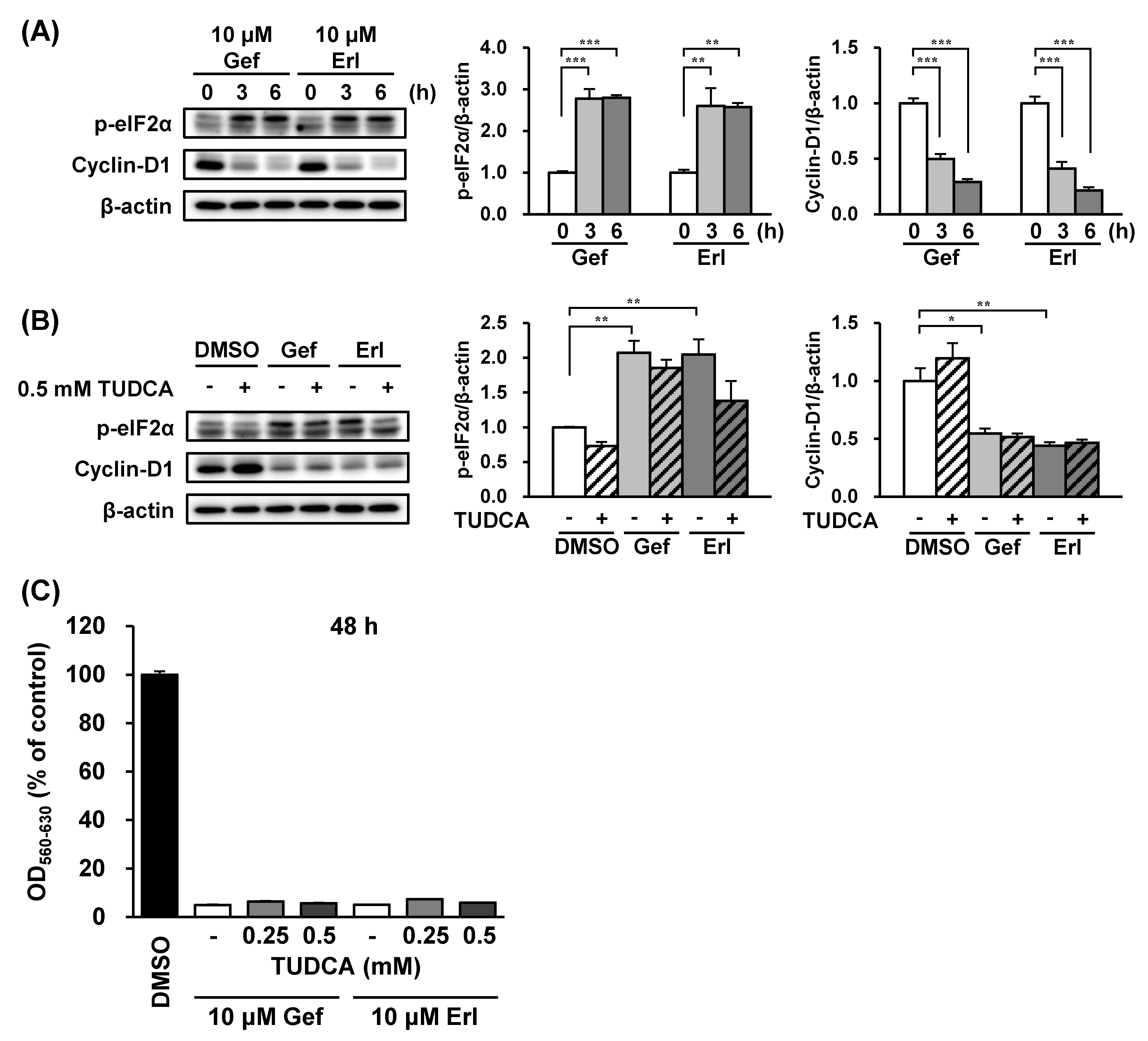

Supplement: S2 Fig — PC-9 cells were treated with gefitinib or erlotinib (10 μM) with/without TUDCA for indicated times (A), 3 h (B) and 48 h (C). Whole-cell lysates were analyzed by immunoblotting using antibodies respectively specific against phosphorylated eIF2α, cyclin-D1 and β-actin. Representative images of three independent experiments are shown. (C) Cell counts were estimated by the MTT assay. Data are expressed as means ± S.E.M. of three independent experiments. Asterisks indicate significant differences between two groups. (*; p < 0.05, **; p < 0.01, ***; p < 0.001, one-way ANOVA with Tukey-Kramer’s tests). (TIFF) [file pone.0136176.s002.tiff]
